# Adherence and clinical outcomes of HIV patients switching to a fixed-dose combination regimen

**DOI:** 10.4102/sajid.v37i1.464

**Published:** 2022-10-24

**Authors:** Geziena E. Kruger-Swanepoel, Martie S. Lubbe, Dorcas M. Rakumakoe, Martine Vorster

**Affiliations:** 1 Medicine Usage in South Africa (MUSA), Faculty of Health Sciences, North-West University, Potchefstroom, South Africa

**Keywords:** HIV, adherence, antiretroviral therapy, fixed-dose combination, Northern Cape, viral load, CD4 count, South Africa

## Abstract

**Background:**

The efficacy of antiretroviral therapy (ART) is monitored using clinical markers such as viral load (VL) and CD4 counts. Adherence to ART has been associated with viral suppression and improved clinical outcomes.

**Objectives:**

To determine the relationship between adherence status with multiple-tablet regimens (MTR) and fixed-dose combination (FDC) regimens, to weight, CD4 count and VL of patients living with HIV.

**Method:**

An observational, descriptive study was conducted on a closed cohort of patients living with HIV and attending a primary health care clinic in Northern Cape in South Africa between 01 January 2013 and 31 December 2015. Patients were on an MTR and changed to an FDC regimen. Adherence was measured using the medicine possession ratio (MPR).

**Results:**

Statistically significant differences exist between the mean MPR of the 30-day (*p* = 0.0308) and 28-day supply (*p* < 0.0001) of the MTR when compared to FDC regimen. No statistically significant differences could be found between adherence and clinical outcomes such as weight, CD4 count and VL for either MTR or FDC regimens. The suppressed VL values measured for MTR were *n* = 299 (89.25%) and *n* = 415 (93.05%) for FDC regimen.

**Conclusion:**

Adherence improved when patients were switched to FDC, but no statistically significant differences in clinical outcomes measured as weight, CD4 count and VL between adherence status and regimen type could be found.

**Contribution:**

This study contributes to much-needed information about ART adherence and clinical outcomes (such as weight, CD4 count and VL) of adult HIV-positive patients in a public healthcare clinic in the Northern Cape, South Africa.

## Introduction

Specific laboratory clinical markers such as viral load (VL) and CD4 lymphocyte counts are used to monitor the clinical efficacy of antiretroviral therapy (ART) in patients living with HIV. The main goal of ART is to suppress and maintain suppression of VL to lower than detectable limits (LDL) and to increase the CD4 lymphocyte count.^1,2,3^ Viral suppression is one of the components of the Joint United Nations Programme on HIV and AIDS (UNAIDS) 95-95-95 targets to be reached by 2030.^4^

Literature explains the association between good adherence and suppressed VL.^2,5,6,7,8,9^ It has been shown that using a fixed-dose combination (FDC) regimen improves virological outcomes and lowers associated healthcare costs.^10^ An ART adherence of more than 95% is required to achieve viral suppression.^7,9^ Patients with residual low-level viraemia are associated with lower adherence, but patients with a moderate adherence can also have a steady viral suppression.^3,7^ Various methods can be used to determine adherence to ART.^11^ The use of pharmacy refill data to calculate adherence by determining the proportion of days covered was found to be predictive of VL rebound in patients already suppressed on ART.^2^

Previously, low- and middle-income countries such as those in sub-Saharan Africa had no access to VL testing. Clinicians relied on clinical assessment and CD4 counts to determine the effectiveness of ART.^12,13^ Later, the World Health Organization (WHO) announced that CD4 counts were unreliable to determine if a patient was failing on a first-line ART regimen.^13,14^ It was discovered that CD4 counts were not always associated with adherence or the treatment success or failure of ART and that patients monitored using only CD4 counts had higher mortality rates.^12,13,15^ Although the CD4 count is unreliable as a measure of virological outcomes, it is still used to determine the immunological status of people living with HIV.^16^ The CD4 count can assist clinicians in assessing the severity of the immune suppression caused by the HIV infection to fast-track ART initiation and determine whether co-trimoxazole preventive therapy is indicated.^17,18^ All patients with a CD4 count of less than 100 cells/µL should be screened for the cryptococcal antigen and the need for antifungal therapy.^14,16,18^

In line with the WHO^19^ recommendations, South Africa incorporated VL testing once a year on patients with a VL of less than 1000 copies/mL.^17^ If a VL is more than 1000 copies/mL, the VL testing must be repeated within three months after adherence was addressed.^18,19^ Virological failure is defined as two consecutive VL values of more than 1000 copies/mL on a nucleoside reverse transcriptase inhibitor-based (NRTI-based) antiretroviral (ARV) regimen.^16,17,18^ Before 2017, the CD4 count was taken as a baseline and then annually to determine immune function and eligibility for ART initiation.^17,20,21^ Guidelines after 2017 incorporated the WHO recommendation of testing and treating all.^16,18^

An FDC regimen is used to simplify complex regimens and decrease the pill burden, facilitating improved adherence to ART.^22,23,24,25^ It was found that adherence improved in women living with HIV who had been switched from a multiple-tablet regimen (MTR) to an FDC regimen.^26^ In 2010, Salami et al.^27^ reported a mean adherence of 70.8% of patients accessing ART services at the University of Ilorin Teaching Hospital in Nigeria. In 2019, a study conducted at the same hospital in Nigeria reported an adherence of 92.6% and attributed the observed increase in the mean adherence to be related to the increased use of FDC regimens.^28^

It has been found that patients initiated on ART experienced weight gain as part of their overall improvement in health.^8,29,30,31,32^ In the clinical setting, weight monitoring is used to assess patients’ responses to ART and detect opportunistic infections such as tuberculosis (TB), which is known to cause sudden weight loss.^14,16,20,31,33^

In this study in a rural primary health care (PHC) clinic in the Northern Cape in South Africa, the researchers determined the relationship between the adherence status on MTR and FDC regimens and clinical outcomes such as VL, CD4 count and weight of patients living with HIV.

## Research methods and design

### Research design and setting

An observational, descriptive study was conducted on a closed cohort of patients living with HIV and attending a PHC clinic in the Frances Baard District of the Northern Cape province of South Africa between 01 January 2013 and 31 December 2015. The patients were on an MTR and changed to an FDC regimen during the study period. The 2010 ARV standard treatment guidelines recommended all patients on a stavudine (D4T) containing regimen showing early signs of toxicity to be switched to tenofovir (TDF).^20^ At this stage, only MTRs were available in the public sector. The updated South African antiretroviral treatment guidelines of 2013 incorporated the use of FDC regimens.^17^ This study investigated the adherence of patients who were switched to an FDC regimen because of the updated changes of the South African antiretroviral treatment guidelines of 2013.

### Study population

**FIGURE 1:**
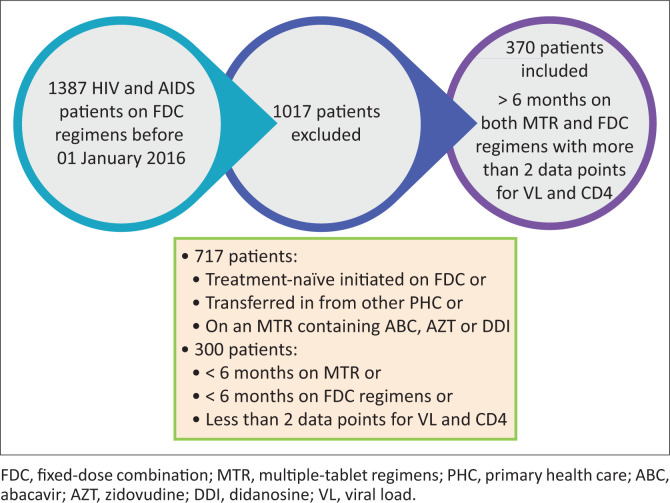
Selection of participants.

**FIGURE 2:**
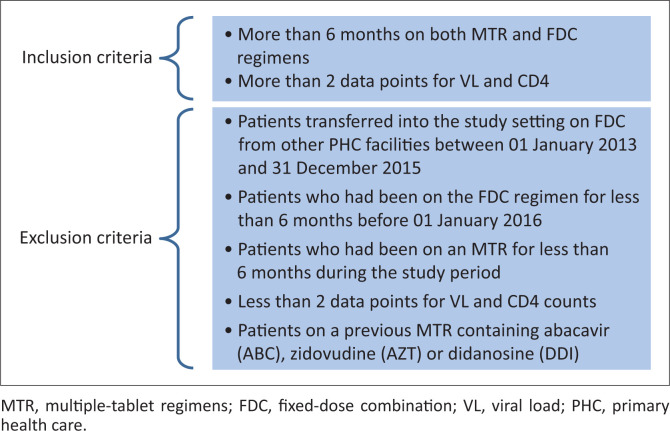
Inclusion and exclusion criteria.

The target population consisted of 1387 patients older than 18 years who were living with HIV, already on ART and already on an FDC regimen until 31 December 2015 (see Figure 1). Only 370 patients complied with the inclusion criteria, had received an MTR for more than six months before being changed to an FDC regimen and had been on the FDC regimen for more than six months during the study period. These patients had at least 2 data points each available for VL and CD4 counts during the study period (see Figure 2). The reason for the inclusion criteria of more than six months each on an MTR and an FDC was to ensure enough data were available to compare the medicine possession ratio (MPR) of each regimen type. Patients on an MTR containing abacavir (ABC), zidovudine (AZT) and didanosine (DDI) were excluded, because it would be implausible for patients on this MTR type to have been swopped to a TDF-containing FDC regimen because of the contraindications associated with TDF, such as renal impairment. See Figure 2 for the detailed inclusion and exclusion criteria.

### Data source and collection process

Retrospective data was collected by means of the iDart dispensing programme (Cell Life, Providence, Rhode Island, United States) and the Tier.net database (Tier.net Technologies LLC, Melbourne, Florida, United States) to identify and select participants according to the inclusion and exclusion criteria (see Figure 2). After the selection of participants, patient files were used to verify the information obtained from the electronic resources using a self-developed survey form. Each participant was assigned a unique number to anonymise the data.

### Development of data collection tools

The iDart dispensing programme was used to collect prescription refill dates and quantities and the type of ARV regimen issued. The Tier.net database was used to verify dates of prescription refills and collect data on CD4 counts and VL and the dates these tests were done. Lastly, patient files were used to verify all the data collected from the iDart and the Tier.net databases and to collect data on patients’ recorded weights and the dates they were taken.

The MTR ARVs were dispensed in different pack sizes depending on the brand issued; it could be either a 28 or a 30-day supply. The dispensing records only indicated that a month’s supply had been given to the patient, not whether it contained a 28 or 30-day supply. To adjust for this limitation, the MPR for the MTR was calculated for both a 28 and 30-day supply. The FDC pack sizes received by the pharmacy during the study period were all for 28 days; therefore, the calculation of the MPR for the FDC regimen did not have this limitation.

### Data analysis

The MPR was calculated using the following equation multiplied by 100 to represent the ratio as a percentage^34,35^:


$$MRP=\frac{Sum\;of\;the\;supplied\;medication\;in\;days}{Number\;of\;days\;in\;refill\;interval}\times 100$$ [Eqn 1]

The different adherence categories used were nonadherent (MPR < 95%), adherent (MPR ≥ 95% < 110%), and oversupply (MPR ≥ 110%).

Data were analysed using the Statistical Package for the Social Sciences (SPSS) version 27 (IBM Corporation, Armonk, New York, United States) and the Statistical Analysis System (SAS) version 9.4 (SAS Institute, Cary, North Carolina, United States) (SAS Institute Inc., 2002–2012).

Variables were expressed using descriptive statistics such as frequencies (*n*), percentages (%), means, medians, standard deviations and 95% confidence intervals (CI). All statistical significance was considered with a two-sided probability of *p* < 0.05. The practical significance of differences was computed when the results were statistically significant (*p* ≤ 0.05).

The two-sample *t-*test was used to determine the statistically significant difference between the mean MPR of the treatment groups (MTR vs. FDC regimens). The chi-square test was used to determine an association between proportions of two or more categorical variables, such as the adherence categories and regimen type. After that, Cramer’s *V* statistic was used to evaluate the practical significance of this association (with Cramer’s *V* ≥ 0.5 defined as practically significant).

A linear mixed model was used to describe the effect of adherence status and regimen type on weight, CD4 counts and VL. An unstructured covariance matrix was used and subject treated as a random effect to take into account the clustered structure of the data and the dependency of the observations. Cohen’s *d*-values were determined when statistically significant (*p* ≤ 0.05) differences in the mean weight, CD4 counts and VL were obtained between patients on MTR and FDC regimens (with *d* ≥ 0.8 defined as a large effect with practical significance).

For the purposes of this study, the VL data were categorised as follows:

- Suppressed viral load:
  ■ lower than the detectable limit (LDL)
  ■ < 20 copies/mL
  ■ < 40 copies/mL
  ■ < 150 copies/mL
  ■ any amount < 400 (because of the limit of the assays used by the labs during the study period).
- Low-level viraemia:
  ■ ≥ 400 < 1000 copies/mL.
- Unsuppressed viral load:
  ■ ≥ 1000 copies/mL.

The input data on the survey form for the VL were copied from the laboratory results in the patient files. For this study, a VL of less than 400 copies/mL would be considered a suppressed VL because of different types of assays used in laboratories to determine VL. When the data were collected, the specificity of the test used for each participant and the sample volume were not indicated. Therefore, the LDL data were conservatively considered to be less than 400 copies/mL.

## Results

### Demographics

The demographic information of the study population (*n* = 370) is summarised in Table 1. There were more female patients (67.49%; *n* = 247) than male patients (32.51%; *n* = 119) in the study population (*N* = 366). The genders of four patients were marked as unknown on the survey tool and reported as missing entries in the data analysis (see Table 1). A total of 20 participants did not have data on either gender or date of birth and were excluded when the mean age of patients (*N* = 350) was calculated. The median age for the study population was 41.36 years (interquartile range [IQR]: 12.56 years) (see Table 1).

**TABLE 1:** Demographic profile and multiple-tablet regimen types.

Variable	Number of patients			
	Total (*N*)		Female (*n*)	Male (*n*)
	*n*	%		
Gender	366†	-	247	119
Age (years)	350†#	-	350†‡	350‡
Median age (years) (*N* = 350‡)	14.36	-	40.23	45.20
IQR	12.56	-	12.46	15.00
**MTR ARV regimen types (*n* = 432)**				
TDF/3TC/EFV200	3	0.69	-	-
TDF/3TC/EFV600	286	66.20	-	-
TDF/3TC/NVP	46	10.65	-	-
d4T/3TC/EFV200	1	0.23	-	-
d4T/3TC/EFV600	82	18.98	-	-
d4T/3TC/NVP	13	3.01	-	-
TDF/3TC/d4T§	1	0.23	-	-

TDF, tenofovir; MTR, multiple-tablet regimens; EFV, efavirenz; NVP, nevirapine; d4T, stavudine; ARV, antiretroviral; IQR, interquartile range.

† , 4 entries missing because gender not filled in for all participants;

‡ , 20 entries missing because of date of birth and gender not filled in for all participants;

§ , TDF/3TC/d4T not a valid regimen; information on data collection tool not completed correctly.

During the study period, 63 patients’ MTR regimen changed because of side effects such as lipoatrophy, in which case a d4T-containing MTR would be switched to a TDF-containing MTR. These patients were counted more than once when the data were statistically analysed. Therefore, the total number of patients who received an MTR (*n* = 432) was more than the actual study population of 370 patients (Table 1). The median time on an MTR regimen was 349.50 days (IQR: 189.00) and on the FDC regimen was 615.00 days (IQR: 78.00).

### Adherence

The adherence status of patients as calculated using MPR is indicated in Table 2. The adherence status was calculated twice in the MTR group to accommodate both a 28-day and a 30-day supply of medication. The MPR for the FDC regimen was only calculated for a 28-day supply. Statistically significant differences were found when the mean MPR of the 28-day and 30-day supply of MTR were compared to the FDC regimen’s mean MPR (*p* < 0.0001 and *p* = 0.0308, respectively) (Table 2). No practically significant difference was found between the mean MPR of the MTR 30-day supply and those of the FDC regimen (*d* = 0.226). A practically significant difference (*d* = 0.939) was found between the mean MPR of the MTR 28-day supply and those of the FDC (Table 2).

**TABLE 2:** Number of patients according to regimen type and adherence.

Adherence categories	Multiple-tablet regimen 28-day supply (*N* = 432)					FDC tablet 28-day supply, (*N* = 369)					*p*	Effect	Multiple-tablet regimen 30-day supply (*N* = 432)					FDC tablet 28-day supply, (*N* = 369)					*p*	Effect
	*n*	%	Mean	s.d.	95% CI	*n*	%	Mean	s.d.	95% CI			*n*	%	Mean	s.d.	95% CI	*n*	%	Mean	s.d.	95% CI		
MPR < 95%	268	62.04	-	-	-	82	22.22	-	-	-	0.5425*	Cramer’s *V* = 0.0599	147	34.03	-	-	-	82	22.22	-	-	-	0.0071*	Cramer’s *V* = 0.1278
MPR ≥ 95% < 110%	152	35.19	-	-	-	273	73.98	-	-	-	-	-	222	51.39	-	-	-	273	73.98	-	-	-	-	-
MPR ≥ 110%	12	2.78	-	-	-	14	3.79	-	-	-	-	-	63	14.58	-	-	-	14	3.79	-	-	-	-	-
MPR (%)	-	-	88.88	17.91	87.19–90.58	99.96	11.25	99.96	11.25	98.45–101.48	< 0.0001**	Cohen’s *d* = 0.939	-	-	96.98	18.77	95.21–98.76	-	-	99.96	11.25	98.45–101.48	0.0308**	Cohen’s *d* = 0.226

MPR, medicine possession ratio; FDC, fixed-dose combination; CI, confidence intervals.

* , The *p*-value was calculated using Pearson’s chi-square test;

** , The *p*-value was calculated using the two-sample *t-*test

A linear mixed-model analysis was used to compare the weight, CD4 count and VL of patients according to adherence status per regimen type.

### Weight

When the mean weight of patients on the MTR 28-day supply and the MTR 30-day supply were compared to the mean weight of patients on the FDC, no statistically significant differences could be found (*p* > 0.05). The mean weight of patients on the MTR 28-day supply was 64.71 kg (95% CI: 61.46–66.87) and on the FDC 65.78 kg (95% CI: 63.10–68.45). No statistically significant difference was found between the mean weight of patients on the MTR 28-day supply and the FDC (*p* = 0.240). The mean weight of patients on the MTR 30-day supply was 65.13 kg (95% CI: 63.02–67.24) and for the FDC 65.83 kg (95% CI: 63.16–68.51). These differences were also not statistically significant (*p* = 0.529).

The mean weight of patients in the different adherence categories was compared with each other for both the 28-day and 30-day supply of MTR. The results were *p* = 0.313 and *p* = 0.103, respectively, and revealed no statistically significant differences.

There was no statistically significant difference in patients’ mean weight between adherence categories and type of regimen when the MPR was calculated using a 28-day supply of MTR (*p* = 0.879) (Table 3). The same trend was found when the MPR was calculated using a 30-day supply of MTR (*p* = 0.654) (Table 3).

**TABLE 3:** The effect of regimen type and adherence categories on weight.

Type of regimen	Adherence category	Mean weight (kg)	95% CI		*p*-value
			Lower bound	Upper bound	
**MTR 28-day supply compared with FDC 28-day supply**					
1. MTR 28-day supply	MPR < 95%	65.73	63.60	67.85	0.879
	MPR ≥ 95% < 110%	64.35	61.99	66.72	
	MPR ≥ 110%	62.42	56.30	68.53	
2. FDC regimen	MPR < 95%	66.55	63.83	69.27	
	MPR ≥ 95% < 110%	65.74	63.73	67.75	
	MPR ≥ 110%	65.04	58.99	71.08	
**MTR 30-day supply compared with FDC 28-day supply**					
1. MTR 30-day supply	MPR < 95%	67.02	64.52	69.52	0.654
	MPR ≥ 95% < 110%	64.44	62.28	66.59	
	MPR ≥ 110%	63.94	60.63	67.24	
2. FDC regimen	MPR < 95%	66.78	64.05	69.51	
	MPR ≥ 95% < 110%	65.63	63.61	67.64	
	MPR ≥ 110%	65.09	59.04	71.14	

Note: Type of regimen and adherence categories of FDC compared with MTR 28-day and MTR 30-day supply.

MTR, multiple-tablet regimens; MPR, medicine possession ratio; FDC, fixed-dose combination; CI, confidence interval.

### CD4 count

The mean CD4 count of patients on the MTR 28-day supply and the MTR 30-day supply was compared with the mean CD4 count of patients on the FDC, and no statistically significant difference could be found (*p* > 0.05) (Table 4). The mean CD4 count for patients on the MTR 28-day supply was 516.30 cells/µL (95% CI: 471.45–561.15) and for the FDC 531.81 cells/µL (95% CI: 489.80–573.81). The mean CD4 count for patients on the MTR 30 days’ supply was 504.03 cells/µL (95% CI: 474.38–533.68) and for the FDC 529.13 cells/µL (95% CI: 487.13–571.14). No statistically significant differences were found between the mean CD4 count of patients on the 28-day (*p* = 0.557) and 30-day supply of MTR (*p* = 0.236) and those of patients on the FDC.

**TABLE 4:** The effect of regimen type and adherence categories on CD4 count.

Type of regimen	Mean CD4 (cells/μL)	95% CI		*p*-value
		Lower bound	Upper bound	
**MTR 28-day supply compared with FDC 28-day supply**				
1. MTR 28-day supply				
MPR < 95%	508.39	479.07	537.71	0.209
MPR ≥ 95% < 110%	505.91	466.95	544.86	
MPR ≥ 110%	534.60	416.45	652.74	
2. FDC regimen				
MPR < 95%	551.40	504.25	598.55	
MPR ≥ 95% < 110%	573.84	546.01	601.67	
MPR ≥ 110%	470.17	363.37	576.98
**MTR 30-day supply compared with FDC 28-day supply**				
1. MTR 30-day supply				
MPR < 95%	504.15	466.70	541.60	0.315
MPR ≥ 95% < 110%	513.49	482.17	544.81	
MPR ≥ 110%	494.45	433.44	555.45	
2. FDC regimen				
MPR < 95%	549.32	501.91	596.73	
MPR ≥ 95% < 110%	574.18	546.34	602.01	
MPR ≥ 110%	463.90	357.27	570.53

Note: Type of regimen and adherence categories of FDC compared with MTR 28-day supply.

MTR, multiple-tablet regimens; MPR, medicine possession ratio; FDC, fixed-dose combination; CI, confidence interval.

The mean CD4 counts of patients on the MTR 28-day supply (*p* = 0.631) and the MTR 30-day supply (*p* = 0.127) in the different adherence categories were compared, and no statistically significant differences were found between adherence categories.

There was no statistically significant difference between adherence categories, type of regimen and CD4 counts when the MPR for the MTR was calculated for the 28-day supply (*p* = 0.209) (Table 4). The same trend was found when the MPR was calculated for the 30-day supply (*p* = 0.315) (Table 4).

### Viral load

The mean VL was also statistically analysed using a linear mixed-model analysis, and no statistically significant differences were found between adherence categories, type of regimen and the mean VL for both the MTR 28-day supply and the MTR 30-day supply (*p* = 0.690 and *p* = 0.378, respectively) (Table 5). A statistically significant difference was found between mean VL and adherence categories when the adherence for the MTR was calculated using a 30-day supply (*p* = 0.045).

**TABLE 5:** The effect of regimen type and adherence categories on viral load.

Type of regimen	Mean VL (copies/mL)	95% CI		*p*
		Lower bound	Upper bound	
**MTR 28-day supply compared with FDC 28-day supply**				
1. MTR 28-day supply				
MPR < 95%	13 805.86	3848.93	23 762.79	0.690
MPR ≥ 95% < 110%	4603.15	−9085.04	18 291.33	
MPR ≥ 110%	3582.11	−44 054.16	51 218.39	
2. FDC regimen				
MPR < 95%	14 945.52	46.25	29 844.80	
MPR ≥ 95% < 110%	7622.18	−82.95	15 327.30	
MPR ≥ 110%	35 338.44	−12 297.83	82 974.72	
**MTR 30-day supply compared with FDC 28-day supply**				
1. MTR 30-day supply				
MPR < 95%	23 328.81	9741.87	36 915.75	0.378
MPR ≥ 95% < 110%	2538.11	−8083.30	13 159.51	
MPR ≥ 110%	10 440.06	−13 998.67	34 878.79	
2. FDC regimen				
MPR < 95%	14 945.52	88.76	29 802.28	
MPR ≥ 95% < 110%	7622.18	−60.97	15 305.32	
MPR ≥ 110%	35 338.44	−12 161.91	82 838.80	

Note: Type of regimen and adherence categories of FDC compared with MTR 28 day supply.

MTR, multiple-tablet regimens; MPR, medicine possession ratio; FDC, fixed-dose combination; CI, confidence interval; VL, viral load.

The lower bound of the 95% CI of the mean VL of some categories showed negative values (see Table 5), which is impossible because of VL copies/mL being strictly positive. These negative lower bounds can be because of the limited number of data points available to analyse VL as continuous data. Therefore, it was decided that the VL results would be presented as categorical data (Table 6) because of the limited number of data points available to analyse the VL as continuous data and determine the difference in mean VL according to adherence and regimen types.

**TABLE 6:** Description of the viral load results before and after switching to a fixed-dose combination regimen.

VL measurements	Number of observations (*n*)	Percentage (%)
**VL measurements of the MTR (before switching)**		
Suppressed < 400 copies/mL	299	89.25
Low-level viraemia ≥ 400 < 1000 copies/mL	20	5.97
Unsuppressed ≥ 1000 copies/mL	16	4.78
Total observations	335	100.00
**VL measurements of the FDC regimen (after switching)**		
Suppressed < 400 copies/mL	415	93.05
Low-level viraemia ≥ 400 < 1000 copies/mL	5	1.12
Unsuppressed ≥ 1000 copies/mL	26	5.83
Total observations	446	100.00

VL, viral load; FDC, fixed-dose combination; MTR, multiple-tablet regimens.

The number of suppressed VLs measured during the study period for the MTR was 299 (89.25%). A total of 36 data points (10.75%) were not suppressed (VL > 400 copies/mL). Of these 36 data points, 20 data points (5.97%) had low-level viraemia and 16 data points (4.78%) had VL > 1000 copies/mL. The number of suppressed VLs measured during the study period for the FDC regimen was 415 (93.05%). A total of 31 data points (6.95%) were not suppressed (VL > 400 copies/mL), of which 5 data points (1.12%) had low-level viraemia and 26 data points (5.83%) had VL > 1000 copies/mL.

## Discussion

Adherence improved when switching from an MTR to an FDC regimen (*p* < 0.05) (Table 2). No statistical differences could be found between the mean weight, CD4 count and VL for the adherence categories of the different regimen types (MTR vs. FDC regimen) (*p* > 0.05). This result could be because both the MTR and FDC regimens consisted of two NNRTIs and one NRTI. It is therefore expected that weight, CD4 and VL will remain the same before and after switching from an MTR to an FDC.

The effect of adherence on weight is not well documented, and the studies that did investigate weight as a study variable mainly did so on ART-naïve patients initiated on ART and compared weight or body mass index (BMI) with virological outcomes.^5,36,37,38^ These study results could not be compared with studies focusing on ART-naïve patients initiated on ART, because the current study population consisted of patients already on ART. The length of the patients was also not available to calculate BMI, for better comparison.

Contradictory to the current study findings, a previous study found that higher CD4 counts and the use of FDC regimens were related to improved adherence.^39^ Also, a low CD4 count was associated with suboptimal adherence when pharmacy refill data were used to measure adherence.^40^ This study did find that using FDC regimens improves adherence. However, the researchers could not find statistically significant differences in the mean CD4 counts according to patient adherence on MTR and FDC regimens (see Table 4). A systematic review done by Clay et al.^24^ was unable to find any differences in the increase of CD4 counts and patients’ adherence to MTR and FDC regimens, which is in line with the findings of this study. According to a systematic review done by Bock et al.,^15^ the relationship between CD4 counts and adherence varies. They were unable to find sufficient evidence to show an association between high and low baseline CD4 counts and differences in adherence of patients initiated on ART.^15^ Baseline CD4 counts were not documented for this study.

Early studies showed that discontinuation of ART had been associated with lower CD4 counts and that adherence of more than 95% is associated with increases in CD4 counts.^9,41^ Salinas et al.^42^ found that the baseline CD4 count was the only laboratory variable in their study that showed a decreased hazard in discontinuation of first-line ART. The median CD4 count in their study population was 95 cells/mL. It was found that patients initiated on ART with higher baseline CD4 counts (early-stage HIV infection with CD4 > 350 cells/µL) were more adherent than those who were initiated on ART with low baseline CD4 counts (late-stage HIV infection with CD4 < 200 cells/µL).^43^ By contrast, Meloni et al.^44^ discovered that the average adherence was lower in patients initiated at higher baseline CD4 counts (> 350 cells/mm^3^). However, they did find that the median CD4 count of the cohort increased over time on ART.^44^ A study by Ehlers and Tshisuyi^45^ conducted in a rural district in Botswana yielded similar results. Patients initiated at lower CD4 counts (< 100 cells/mm^3^) showed higher adherence, which indicates a marginal association between adherence and CD4 counts (*p* = 0.046).^45^

The data shows that, over the study period, viral suppression was achieved in 89.25% of the measurements on the MTR and 93.05% of measurements on the FDC regimen. However, the clustered structure of the data must be taken into consideration; therefore, the measurements cannot be representative of the total study population.

Patients with low-level viraemia are at a higher risk of developing resistance to ART, especially if the regimen is NRTI-based (as all the participants in this study were on regimens containing either nevirapine [NVP] or efavirenz [EFV]).^46^ This study showed that 5.97% of the VL measurements of patients on the MTR and 1.12% of the VL in patients on the FDC regimen showed low-level viraemia. In patients with optimal adherence, an unsuppressed VL can either be because of nonadherence, low ARV dosage, drug–drug interactions or high levels of ARV-resistant mutations.^3,46^ Viral load can also be used as a tool to distinguish between poor adherence and resistance to ART, as patients with poor adherence will most likely have a suppressed VL after an adherence intervention.^16^

### Strengths and limitations

The data were collected retrospectively and verified using three datasets (iDart dispensing programme, Tier.net database and the patients’ files), strengthening the validity of the collected data.

The retrospective nature of this study provides insight into a real-world setting. This study contributes to the much-needed information about ART adherence and clinical outcomes (such as weight, CD4 count and VL) of adult HIV-positive patients of a public healthcare clinic in a rural part of the Northern Cape in South Africa.

Collecting data retrospectively from clinical files can have limitations, such as incomplete data because of negligent capturing or an inattentive clinician. To compensate for one identified limitation, the MPR of the MTR group was calculated twice – for a 28-day supply and a 30-day supply – as the exact quantities dispensed to the patients were not documented. Using pharmacy refill data and MPR to calculate adherence has its limitations, as it must be assumed that the medication dispensed was correctly taken by the patient.^11,34,47,48^ Unfortunately, MPR calculated from pharmacy refill data cannot give information on how and when patients took their medication.^47^

Another limitation to this study was the limited number of data points for CD4 count and VL, which restricted the use of inferential statistics. Thus, only descriptive statistics could be used for VL values. Possible reasons for the lack of data points could be that results were never documented, blood was never drawn or it was not drawn timeously by the clinician according to the set guidelines. In future studies, researchers should try to include more data points for CD4 counts and VL per regimen type for each participant. As the same group of patients’ clinical data were compared before and after switching to an FDC regimen, this might have influenced the results of the study.

The researcher only collected data from one rural clinic in the Northern Cape province; therefore, the results may not apply to all rural clinics in the Northern Cape. The results may look different if data from more rural clinics in the Northern Cape were included.

### Implications or recommendations

The implication for clinical practice is that FDC improves adherence. This study helps to emphasise the importance of developing new FDC, even for more complex ARV regimens, to help improve patients’ adherence, specifically in rural areas of the Northern Cape province.

Further studies are necessary to investigate the relationship between clinical outcomes such as VL and CD4 count and adherence in patients who attend rural PHC clinics in the Northern Cape. Studies could be extended over longer periods of time and include larger study populations to better understand the influence of adherence on clinical outcomes.

## Conclusion

In conclusion, this study did find that switching from an MTR to an FDC improves adherence to ART. No statistically significant differences could be found between adherence and clinical outcomes such as weight, CD4 count and VL. More research is required on larger study populations over longer periods of time in the public healthcare environment in rural areas.

## References

[1] Boillat-Blanco N, Darling KEA, Schoni-Affolter F, et al. Virological outcome and management of persistent low-level viraemia in HIV-1-infected patients: 11 years of the Swiss HIV cohort study. Antivir Ther. 2015;20(2):165–175. 10.3851/IMP2815 24964403

[2] Cambiano V, Lampe FC, Rodger AJ, et al. Use of a prescription-based measure of antiretroviral therapy adherence to predict viral rebound in HIV-infected individuals with viral suppression. HIV Med. 2010;11(3):216–224. 10.1111/j.1468-1293.2009.00771.x 20002781

[3] Widera M, Dirks M, Bleekmann B, et al. HIV-1 persistent viremia is frequently followed by episodes of low-level viremia. Med Microbiol Immunol. 2017;206(3):203–215. 10.1007/s00430-017-0494-1 28220254 PMC5409919

[4] Joint United Nations Programme on HIV/AIDS (UNAIDS). Fast-Track – Ending the AIDS epidemic by 2030 [homepage on the Internet]. 2014 [cited 2021 Nov 15]. Available from: https://www.unaids.org/sites/default/files/media_asset/JC2686_WAD2014report_en.pdf

[5] Hong SY, Jerger L, Jonas A, et al. Medication possession ratio associated with short-term virologic response in individuals initiating antiretroviral therapy in Namibia. PLoS One. 2013;8(2):e56307. 10.1371/journal.pone.0056307 23509605 PMC3585291

[6] Howard AA, Arnsten JH, Lo Y, et al. A prospective study of adherence and viral load in a large multi-center cohort of HIV-infected women. AIDS. 2002;16(16):2175–2182. 10.1097/00002030-200211080-00010 12409739

[7] Maggiolo F, Filippo ED, Comi L, et al. Reduced adherence to antiretroviral therapy is associated with residual low-level viremia. Pragmat Obs Res. 2017;8:91–97. 10.2147/POR.S127974 28603436 PMC5457149

[8] Paintsil E. Monitoring antiretroviral therapy in HIV-infected children in resource-limited countries: A tale of two epidemics. AIDS Res Treat. 2011;2011:280901. 10.1155/2011/280901 21490777 PMC3066553

[9] Paterson DL, Swindells S, Mohr J, et al. Adherence to protease inhibitor therapy and outcomes in patients with HIV infection. Ann Intern Med. 2000;133(1):21–30. 10.7326/0003-4819-133-1-200007040-00004 10877736

[10] Homar F, Lozano V, Martínez-Gómez J, et al. Cost analysis of HIV treatment and drug-related adverse events when fixed-dose combinations of antiretrovirals (FDCs) were stopped, versus continuation with FDCs. Health Econ Rev. 2012;3(1):16. 10.1186/2191-1991-2-16 PMC3484113 22943676

[11] Bjarnadóttir MV, Malik S, Onukwugha E, Gooden T, Plaisant C. Understanding adherence and prescription patterns using large-scale claims data. Pharmacoeconomics. 2016;34(2):169–179. 10.1007/s40273-015-0333-4 26660349

[12] Keiser O, Chi BH, Gsponer T, et al. Outcomes of antiretroviral treatment in programmes with and without routine viral load monitoring in southern Africa. AIDS. 2011;25(14):1761–1769. 10.1097/QAD.0b013e328349822f 21681057 PMC3605707

[13] Sigaloff KCE, Hamers RL, Menke J, et al. Early warning indicators for population-based monitoring of HIV drug resistance in 6 African countries. Clin Infect Dis. 2012;54(Suppl 4):S294–S299. 10.1093/cid/cir1015 22544190

[14] World Health Organization. Guidelines for managing advanced HIV disease and rapid initiation of antiretroviral therapy. Geneva: World Health Organization; 2017. 29341560

[15] Bock P, James A, Nikuze A, et al. Baseline CD4 count and adherence to antiretroviral therapy: A systematic review and meta-analysis. J Acquir Immune Defic Syndr. 2016;73(5):514–521. 10.1097/QAI.0000000000001092 27851712

[16] World Health Organization. Consolidated guidelines on the use of antiretroviral drugs for treating and preventing HIV infection: Recommendations for a public health approach. 2nd ed. Geneva: World Health Organization; 2016. 27466667

[17] Republic of South Africa. National Department of Health. The South African antiretroviral treatment guidelines 2013 [homepage on the Internet]. 2013 [updated 2013 Mar 14; cited 2021 Oct 16]. Available from: https://sahivsoc.org/Files/2013%20ART%20Guidelines-Short%20Combined%20FINAL%20draft%20guidelines%2014%20March%202013.pdf

[18] Republic of South Africa. National Department of Health. 2019 ART clinical guidelines for the management of HIV in adults, pregnancy, adolescents, children, infants and neonates [homepage on the Internet]. 2019 [updated 2020 Mar; cited 2021 Sep 29]. Available from: https://sahivsoc.org/Files/2019%20ART%20Guideline%2028042020%20pdf.pdf

[19] World Health Organization. Consolidated guidelines on the use of antiretroviral drugs for treating and preventing HIV infection: Recommendations for a public health approach. Geneva: World Health Organization; 2013. 24716260

[20] Republic of South Africa. National Department of Health. Clinical guidelines for the management of HIV and AIDS in adults and adolescents [homepage on the Internet]. 2010 [updated 2010 Nov 10; cited 2021 Oct 16]. Available from: https://sahivsoc.org/Files/Clinical_Guidelines_for_the_Management_of_HIV_AIDS_in_Adults_Adolescents_2010.pdf

[21] Republic of South Africa. National Department of Health. National consolidated guidelines for the prevention of mother-to-child transmission of HIV (PMTCT) and the management of HIV in children, adolescents and adults [homepage on the Internet]. 2015 [updated 2015 Jun 4; cited 2021 Oct 16]. Available from: https://sahivsoc.org/Files/Consolidated%20ART%20guidelines%20_Jan%202015.pdf

[22] Aldir I, Horta A, Serrado M. Single-tablet regimens in HIV: Does it really make a difference? Curr Med Res Opin. 2014;30(1):89–97. 10.1185/03007995.2013.844685 24040862

[23] Clay PG, Nag S, Graham CM, Narayanan S. Meta-analysis of studies comparing single and multi-tablet fixed dose combination HIV treatment regimens. Medicine. 2015;94(42):e1677. 10.1097/MD.0000000000001677 26496277 PMC4620781

[24] Clay PG, Yuet WC, Moecklinghoff CH, et al. A meta-analysis comparing 48-week treatment outcomes of single and multi-tablet antiretroviral regimens for the treatment of people living with HIV. AIDS Res Ther. 2018;15:17. 10.1186/s12981-018-0204-0 30373620 PMC6206661

[25] Rwagitinywa J, Lapeyre-Mestre M, Bourrel R, Montastruc JL, Sommet A. Comparison of adherence to generic multi-tablet regimens vs. brand multi-tablet and brand single-tablet regimens likely to incorporate generic antiretroviral drugs by breaking or not fixed-dose combinations in HIV-infected patients. Fundam Clin Pharmacol. 2018;32(4):450–458. 10.1111/fcp.12363 29505661

[26] Hanna DB, Hessol NA, Golub ET, et al. Increase in single-tablet regimen use and associated improvements in adherence-related outcomes in HIV-infected women. J Acquir Immune Defic Syndr. 2014;65(5):587–596. 10.1097/QAI.0000000000000082 24326606 PMC3999284

[27] Salami AK, Fadeyi A, James O, Desalu O. Factors influencing adherence to antiretroviral medication in Ilorin, Nigeria. J Int Assoc Provid AIDS Care. 2010;9(3):191–195. https://doi.org/10.1177%2F1545109710368722 10.1177/1545109710368722 20530474

[28] Anyaike C, Atoyebi OA, Musa OI, et al. Adherence to combined antiretroviral therapy (cART) among people living with HIV/AIDS in a tertiary hospital in Ilorin, Nigeria. Pan Afr Med J. 2019;32:e10. 10.11604/pamj.2019.32.10.7508 PMC6497984 31080546

[29] Asemahagn MA, Aragaw A, Agumas Y, et al. Perceived risk factors of HIV infection and ART adherence at Zewditu Memorial Hospital, Addis Ababa, Ethiopia: A survey of people living with HIV/AIDS experiences. Ann Med Health Sci Res. 2018;8(1):105–110.

[30] Galárraga O, Genberg BL, Martin RA, Barton Laws M, Wilson IB. Conditional economic incentives to improve HIV treatment adherence: Literature review and theoretical considerations. AIDS Behav. 2013;17(7):2283–2292. 23370833 10.1007/s10461-013-0415-2 PMC3688660

[31] Meintjes G, Conradie J, Black F, et al. Adult antiretroviral therapy guidelines 2014. S Afr J HIV Med. 2014;15(4):121–143. 10.4102/sajhivmed.v15i4.330

[32] Ware NC, Idoko J, Kaaya S, et al. Explaining adherence success in sub-Saharan Africa: An ethnographic study. PLoS Med. 2009;6(1), e1000011. 10.1371/journal.pmed.1000011 PMC2631046 19175285

[33] Department of Health and Human Services. Guidelines for the use of antiretroviral agents in HIV-1-infected adults and adolescents [homepage on the Internet]. 2013 [updated 2013 Feb 12; cited 2021 Nov 16]. Available from: https://clinicalinfo.hiv.gov/sites/default/files/guidelines/archive/AdultandAdolescentGL003371.pdf

[34] Slabbert FN, Harvey BH, Brink CB, Lubbe MS. Prospective analysis of the medicine possession ratio of antidepressants in the private health sector of South Africa, 2006–2001. S Afr Med J. 2015;105(2):139–144. 26242534 10.7196/samj.8394

[35] Slabbert FN, Harvey BH, Brink CB, Lubbe MS. The impact of HIV/AIDS on compliance with antidepressant treatment in major depressive disorder: A prospective study in a South African private healthcare cohort. AIDS Res Ther. 2015;12:9. 10.1186/s12981-015-0050-2 26261459 PMC4397684

[36] Hoffmann CJ, Charalambous S, Sim J, et al. Viremia, resuppression, and time to resistance in Human Immunodeficiency Virus (HIV) subtype C during first-line antiretroviral therapy in South Africa. Clin Infect Dis. 2009;49(12):1928–1935. 10.1086/648444 19911963 PMC2789416

[37] Messou E, Chaix M, Gabillard D, et al. Association between medication possession ratio, virologic failure and drug resistance in HIV-1 infected adults on antiretroviral therapy in Côte d’Ivoire. J Acquir Immune Defic Syndr. 2011;56(4):356–364. 10.1097/QAI.0b013e3182084b5a 21191309 PMC3050083

[38] Orrell C, Bangsberg DR, Badri M, Wood R. Adherence is not a barrier to successful antiretroviral therapy in South Africa. AIDS [serial online]. 2003 [cited 2021 Oct 24];2003;17(9):1369–1375. Available from: https://journals.lww.com/aidsonline/Fulltext/2003/06130/Adherence_is_not_a_barrier_to_successful.11.aspx 10.1097/00002030-200306130-00011 12799558

[39] Sterrantino G, Santoro L, Bartolozzi D, Trotta M, Zaccarelli M. Self-reported adherence supports patient preference for the single tablet regimen (STR) in the current cART era. Patient Prefer Adherence. 2012;6:427–433. 10.2147/PPA.S31385 22723727 PMC3379866

[40] Mekuria LA, Prins JM, Yalew AW, Sprangers MAG, Nieuwkerk PT. Sub-optimal adherence to combination antiretroviral therapy and its associated factors according to self-report, clinician-recorded and pharmacy-refill assessment methods among HIV-infected adults in Addis Ababa. AIDS Care. 2017;29(4):428–435. 10.1080/09540121.2016.1234681 27701908

[41] Grant LA, Silverberg MJ, Palacio H, et al. Discontinuation of potent antiretroviral therapy: Predictive value of and impact on CD4 cell counts and HIV RNA levels. AIDS. 2001;15(16):2101–2108. 10.1097/00002030-200111090-00005 11684929

[42] Salinas JL, Alave JL, Westfall AO, et al. Medication possession ratio predicts antiretroviral regimens persistence in Peru. PLoS One. 2013;8(10):e76323. 10.1371/journal.pone.0076323 24098475 PMC3788135

[43] Haberer JE, Bwana BM, Orrell C, et al. ART adherence and viral suppression are high among most non-pregnant individuals with early-stage, asymptomatic HIV infection: An observational study from Uganda and South Africa. J Int AIDS Soc. 2019;22(2):e25232. 10.1002/jia2.25232 30746898 PMC6371013

[44] Meloni ST, Chang CA, Eisen G, et al. Long-term outcomes on antiretroviral therapy in a large scale-up program in Nigeria. PLoS One. 2016;11(10):e0164030. 10.1371/journal.pone.0164030 27764094 PMC5072640

[45] Ehlers VJ, Tshisuyi ET. Adherence to antiretroviral treatment by adults in a rural area of Botswana. Curationis. 2015;38(1):1255. 10.4102/curationis.v38i1.1255 PMC6091787 26244453

[46] Briggs R, Templeton K, Fernando I. Comparing HIV viral load assays and frequency of low level virological rebound in clinical practice. Int J STD AIDS. 2014;25(14):1029–1034. 10.1177/0956462414528313 24648315

[47] Arnet I, Abraham I, Messerli M, Hersberger KE. A method for calculating adherence to polypharmacy from dispensing data records. Int J Clin Pharm. 2014;36:192–201. 10.1007/s11096-013-9891-8 24293284 PMC3890044

[48] Kalsekar ID, Madhavan SS, Amonkar MM, et al. Depression in patients with type 2 diabetes: Impact on adherence to oral hypoglycemic agents. Ann Pharmacother. 2006;40(4):605–611. 10.1345/aph.1G606 16551768

